# Construction and validation of a risk prediction model for postoperative ICU admission in patients with colorectal cancer: clinical prediction model study

**DOI:** 10.1186/s12871-024-02598-3

**Published:** 2024-07-04

**Authors:** Lu Wang, Yanan Wu, Liqin Deng, Xiaoxia Tian, Junyang Ma

**Affiliations:** Department of Anesthesia and Perioperative Medicine, General Hospital of Ningxia Medical University, 804 Shengli South Street, Xingqing District, Yinchuan City, Ningxia China

**Keywords:** Intensive care unit, Predictive model, Nomogram, Radical colorectal cancer surgery, Risk factors

## Abstract

**Background:**

Transfer to the ICU is common following non-cardiac surgeries, including radical colorectal cancer (CRC) resection. Understanding the judicious utilization of costly ICU medical resources and supportive postoperative care is crucial. This study aimed to construct and validate a nomogram for predicting the need for mandatory ICU admission immediately following radical CRC resection.

**Methods:**

Retrospective analysis was conducted on data from 1003 patients who underwent radical or palliative surgery for CRC at Ningxia Medical University General Hospital from August 2020 to April 2022. Patients were randomly assigned to training and validation cohorts in a 7:3 ratio. Independent predictors were identified using the least absolute shrinkage and selection operator (LASSO) and multivariate logistic regression in the training cohort to construct the nomogram. An online prediction tool was developed for clinical use. The nomogram's calibration and discriminative performance were assessed in both cohorts, and its clinical utility was evaluated through decision curve analysis (DCA).

**Results:**

The final predictive model comprised age (*P* = 0.003, odds ratio [OR] 3.623, 95% confidence interval [CI] 1.535–8.551); nutritional risk screening 2002 (NRS2002) (*P* = 0.000, OR 6.129, 95% CI 2.920–12.863); serum albumin (ALB) (*P* = 0.013, OR 0.921, 95% CI 0.863–0.982); atrial fibrillation (*P* = 0.000, OR 20.017, 95% CI 4.191–95.609); chronic obstructive pulmonary disease (COPD) (*P* = 0.009, OR 8.151, 95% CI 1.674–39.676); forced expiratory volume in 1 s / Forced vital capacity (FEV1/FVC) (*P* = 0.040, OR 0.966, 95% CI 0.935–0.998); and surgical method (*P* = 0.024, OR 0.425, 95% CI 0.202–0.891). The area under the curve was 0.865, and the consistency index was 0.367. The Hosmer–Lemeshow test indicated excellent model fit (*P* = 0.367). The calibration curve closely approximated the ideal diagonal line. DCA showed a significant net benefit of the predictive model for postoperative ICU admission.

**Conclusion:**

Predictors of ICU admission following radical CRC resection include age, preoperative serum albumin level, nutritional risk screening, atrial fibrillation, COPD, FEV1/FVC, and surgical route. The predictive nomogram and online tool support clinical decision-making for postoperative ICU admission in patients undergoing radical CRC surgery.

**Trial registration:**

Despite the retrospective nature of this study, we have proactively registered it with the Chinese Clinical Trial Registry. The registration number is ChiCTR2200062210, and the date of registration is 29/07/2022.

**Supplementary Information:**

The online version contains supplementary material available at 10.1186/s12871-024-02598-3.

## Introduction

Colorectal cancer (CRC) is the world's second leading cause of cancer-related mortality. The average age of onset is 69 years [1], and the prevalence of the disease is rising [2]. The preferred treatment for CRC is radical resection [3]. However, elderly patients with CRC who often have additional comorbidities are prone to develop postoperative complications, resulting in prolonged hospitalization, increased hospitalization costs, delayed adjuvant treatment, and increased mortality [4]. In Canada, the postoperative transfer rate of CRC patients to the intensive care unit (ICU) postoperatively reaches 17.7%, ranking highest among non-cardiac surgeries [5]. Our preliminary investigation found that 60% of patients undergoing curative CRC surgery and subsequent ICU admission were elderly. However, patients who are admitted to the ICU after surgery for CRC are not always in a critical condition, with many being admitted only for monitoring, placing an unnecessary strain on ICU resources [6]. Additionally, patients in need of intensive care may be overlooked, resulting in deteriorating conditions in regular hospital wards [7]. Thus, timely identification of patients likely to necessitate mandatory ICU admission ensures effective treatment.

As is widely acknowledged, the ICU represents a finite and costly resource, with over 300 million surgeries performed worldwide annually, of which approximately 9.6% of patients require postoperative ICU admission [8, 9]. In China, there is a serious shortage of ICU beds, with only 3.43 ICU beds per 100 000 people [10]. Studies have found that postoperative ICU support for critically ill patients reduces mortality [11]. The most common complications associated with postoperative ICU admission are infection, especially infections of the respiratory system and surgical site [5]. Evidence suggests that ICU stays are costly, strain hospital resources, and result in poor outcomes [12–14]. To date, few reports have addressed the associations between preoperative and intraoperative factors and ICU transfer after CRC resection. There is no risk prediction model available for guidance on whether patients with CRC should be transferred to the ICU after radical resection.

The objective of this retrospective study was to create a predictive nomogram that included a variety of preoperative and intraoperative factors. This nomogram would assist in the early identification and timely management of patients who require mandatory ICU admission immediately following surgery. It focused on preoperative cardiopulmonary function-related indicators, nutritional status, frailty, and intraoperative risk factors. We hypothesized that this predictive model offers a comprehensive reflection of the perioperative position of patients and accurately predicts the risk factors for patients with CRC. Understanding the risks associated with ICU admission after surgery can help anesthesiologists and surgeons reduce unnecessary ICU transfers, thus optimizing medical resource allocation, enhancing care quality, and promoting patient recovery.

## Methods

### Study design

The purpose of this predictive nomogram development study was to construct and validate a nomogram to predict patients most likely to require immediate ICU admission after radical resection of CRC. This study aimed to construct and validate a nomogram for predicting patients at high risk of requiring mandatory ICU admission immediately following radical CRC resection. Construction and validation required preoperative and intraoperative predictive factors, which were obtained by conducting a retrospective survey of patients who had undergone radical or palliative surgery for CRC in the General Hospital of Ningxia Medical University (see below under “Sample”). The General Hospital of Ningxia Medical University is a comprehensive, tertiary, and first-class public teaching hospital in northwest China. This study used the Transparent Reporting of Multivariate Predictive Models for Individual Prognosis or Diagnosis (TRIPOD) (IBM Predictive Analytics, Armonk, NY, USA) [15].

### Study sample and data source

A total of 1005 patients with CRC who underwent radical or palliative surgery for colorectal cancer from August 2020 to April 2022 in Ningxia Medical University General Hospital were included for screening. Patient data from the patient database of Ningxia Medical University General Hospital were reviewed. The Research Ethics Committee of the General Hospital of Ningxia Medical University approved the establishment of this database. Patient records were de-identified and anonymized before analysis. The inclusion criteria were: 1) Histologically confirmed CRC; 2) Patients who underwent radical resection or palliative surgery for CRC. The exclusion criteria were: 1) Presence of colorectal stoma or exploration; 2) Local endoscopic polypectomy; 3) Emergency surgery; 4) Surgery stopped after entering the operating room.

### Ethical considerations

The research protocol was approved by the Ethics Committee of Ningxia Medical University General Hospital (approval number KYLL-2021–1045) and was registered in the China Clinical Trial Registry (ChiCTR2200062210). Because all patient data in the hospital database were de-identified and anonymized before analysis, informed consent of the included patients was waived.

### Indications for postoperative mandatory ICU admission

Indications for postoperative mandatory ICU admission was defined as the presence of one or more of the following features: maintenance of controlled ventilation, reintubation, acute respiratory failure, hemodynamic instability, shock, use of multiple vasoactive drugs and cardiac arrhythmias, as previously described [6, 7].

In this study, postoperative patients were classified into two main groups. The first group included patients who required mandatory ICU admission after surgery, involving the following scenarios: (1) Planned ICU admission for treatment: immediate postoperative ICU admission for patients meeting the criteria for mandatory ICU admission; (2) Unplanned ICU admission within three days after surgery; these were patients who were not admitted to the ICU immediately but required ICU admission within three days of surgery due to acute respiratory failure or other emergent reasons requiring intensive care.

The second category comprised patients who did not require postoperative ICU admission and included the following scenarios: (3) Planned admission to the ICU for monitoring, involving immediate postoperative ICU admission but without meeting the criteria for mandatory ICU admission. These patients were admitted to the ICU solely for monitoring purposes and were transferred to a general ward on the second postoperative day; (4) Discharge without complications: immediate transfer to a general ward after surgery and discharge without any complications.

Lastly, a further scenario was included: (5) Unplanned ICU admission beyond three days after surgery due to delayed complications. These patients were excluded from the analysis as it was difficult to ascertain whether direct ICU admission after CRC surgery could improve patient prognosis.

### Predictors

Data collected from each patient's medical records included: demographic information (age, sex, body mass index [BMI]), Charlson comorbidity index (CCI), American Society of Anesthesiologists (ASA) status, modified frailty index (MFI), and nutritional risk screening 2002 (NRS 2002), and medical history (hypertension, coronary heart disease, percutaneous coronary intervention, atrial fibrillation [AF], chronic obstructive pulmonary disease [COPD], cerebrovascular disease, smoking, alcohol abuse). Laboratory parameters (hemoglobin, serum albumin [ALB], blood urea nitrogen, blood creatinine), respiratory function tests (arterial partial pressure of oxygen, forced vital capacity, forced expiratory volume in one second, peak expiratory flow), cardiac function assessments (left ventricular ejection fraction, fractional shortening), surgical details (tumor location, surgical method, Tumor, Node, Metastasis stage [TNM stage]) and anesthesia factors (operation time, anesthesia time, volume of crystalloid fluid, volume of colloid fluid, urine volume, blood loss, and red blood cell transfusion) were also recorded.

The ASA grade was obtained from the anesthesia record sheet provided and determined by the anesthetist. NRS 2002 was obtained from the medical records. Each admitted CRC patient was routinely assessed by the physician in charge of nutritional risk screening and recorded in the medical records. Since CCI and MFI were not directly recorded in the medical records, a specialized medical researcher summarized them based on the relevant assessment content provided by the medical records. Diagnoses of chronic pulmonary disease, heart disease, hypertension, coronary heart disease, percutaneous coronary intervention, atrial fibrillation, chronic obstructive pulmonary disease, and cerebrovascular disease were made by physicians and recorded in patient records. All laboratory tests and inspection results were the most recent within seven days before surgery. Blood transfusion was indicated for hemoglobin levels < 80 g/L, and for patients with hemoglobin between 80 and 100 g/L; transfusion was based on risk factors associated with hemodynamic instability and inadequate oxygenation [16].

The surgery was performed by experienced surgeons following the clinical practice guidelines of the National Comprehensive Cancer Network (NCCN) [17]. Specimens were collected intraoperatively for routine frozen section analysis. Radical surgery for CRC encompassed both early and intermediate stages. Palliative surgery was only indicated for patients with distant metastases and severe complications of CRC, such as intestinal obstruction, which cannot be resected, or for patients where cancer residue may be present at the resection margins.

### Sample size for model derivation

The minimum sample size was estimated using 36 candidate predictive parameters to establish a binary outcome multivariable prediction model based on the findings of preliminary investigations, assuming an incidence rate of 0.230 (23.0%) and a C-statistic of 0.89 from an existing predictive model. By applying Riley et al.'s method, the estimated minimum sample size for developing the new model was determined to be 874, with 202 positive events of postoperative ICU admission [18].

### Imputation of missing data

Before data analysis, a missing value check was performed on the primary forecast data. A multiple imputation procedure was developed 15 times for incorporation into the study, and a suitable imputation dataset was generated for the final analysis. Multiple imputations deal effectively with missing data and minimize bias by excluding such patients. Furthermore, various imputations still work even when the proportion of missing data is large [19].

### Statistical analysis

All statistical analyses were conducted using R software (Version 4.1.1; https://www.r-project.org) and SPSS 26.0 (SPSS®, Chicago, II, USA). Continuous predictors were presented as mean ± standard deviation (SD), while categorical predictors were presented as numbers and percentages. Pre- and post- imputation datasets were compared using the Kruskal–Wallis non-parametric rank sum test for non-normal distribution. The dataset was randomly divided into a training cohort and a validation cohort in a 7:3, respectively.The dataset was randomly divided into training and validation cohorts in a 7:3 ratio. The training cohort was utilized for model development, while the validation cohort was used for internal validation, retrospectively.To address multicollinearity among predictors, the Least Absolute Shrinkage and Selection Operator (LASSO) method was used to screen out the optimal variables with non-zero coefficients as risk factors at the minimum standard error, as previously described [20]. Then, based on the results of the LASSO regression analysis, independent predictors (*P* < 0.05) were identified using multivariate logistic regression. A nomogram was drawn using the data predicting the occurrence of postoperative special ICU admission. The nomogram's prediction line was used to determine points, which were then summed on the "Total Score" axis to predict the likelihood of postoperative ICU admission on a scale. The Hosmer–Lemeshow test and the coefficient of determination (R2) were used to evaluate the model’s goodness of fit. Discriminative ability was evaluated using the receiver operating characteristic (ROC) curve, area under the ROC curve (AUC), and consistency index (C index). Calibration curves assessed predictive model consistency. Decision curve analysis (DCA) reflected the net benefit of the model for patients. All statistical tests were two-sided; *P* value < 0.05 was considered statistical significance.

## Results

### Study population

**Fig. 1:**
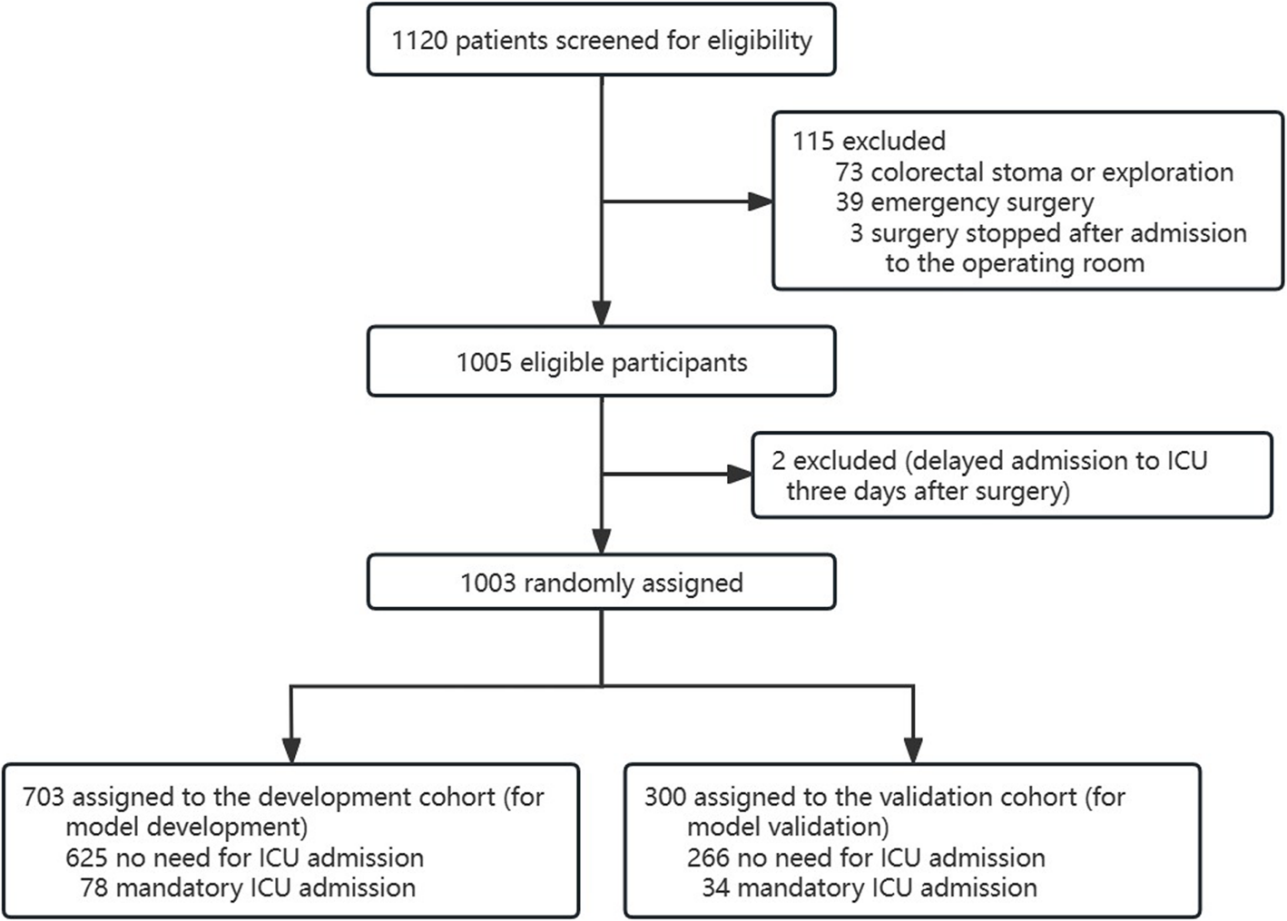
Patients’ flowchart. ICU, intensive care unit

Figure 1 depicts the flow chart for case selection. Between August 2020 and April 2022, 1132 patients underwent retrospective eligibility screening. Of these, 129 patients were excluded: 54 underwent colostomy or exploration, 38 underwent local endoscopic polypectomy, 33 underwent emergency surgery, two were stopped after entering the operating room, and two were delayed. The final analysis comprised 1003 patients, randomized (7:3) into a training cohort (*n* = 703) and a validation cohort (*n* = 300). Of the 703 patients in the training cohort, 625 did not need ICU admission, and the remaining 112 were mandatory admitted to the ICU. Of the 300 patients in the validation cohort, 266 did not need ICU admission, and the remaining 34 were mandatory admitted to ICU. Demographics, pre-existing diseases, laboratory indicators, risk factors related to cardiac function and respiratory examination, and risk factors related to surgical anesthesia were clinically comparable between the two cohorts, as shown in Table 1.

**Table 1:** Baseline characteristics of all patients in the training cohort and validation cohort

Characteristic		Training dataset (*n* = 703)	Validation dataset (*n* = 300)	*P* value
ICU admission		78 (11.1%)	34 (11.3%)	0.999
Age, years	≤ 64	345 (49.1%)	160 (53.3%)	0.251
	65–74	234 (33.3%)	99 (33.0%)	
	≥ 75	124 (17.6%)	41 (13.7%)	
Gender	Male	403 (57.3%)	192 (64.0%)	0.057
	Female	300 (42.7%)	108 (36.0%)	
BMI, kg m^−2^		23.44 (21.5, 25.6)	23.67 (22.0, 25.5)	0.198
CCI	< 3	662 (94.2%)	275 (91.7%)	0.144
	≥ 3	41 (5.8%)	25 (8.3%)	
ASA	II	372 (52.9%)	154 (51.3%)	0.913
	III	324 (46.1%)	143 (47.7%)	
	IV	7 (1%)	3 (1%)	
mFI	Mild	578 (82.2%)	239 (79.7%)	0.407
	Moderate	117 (16.6%)	55 (18.3%)	
	Severe	8 (1.1%)	6 (2%)	
NRS2002	< 3	463 (65.9%)	189 (63%)	0.425
	≥ 3	240 (34.1%)	111 (37%)	
Hypertension	-	481 (68.4%)	195 (65.2%)	0.359
	+	222 (31.6%)	104 (34.8%)	
Coronary heart disease	-	611 (86.9%)	257 (85.7%)	0.668
	+	92 (13.1%)	43 (14.3%)	
PCI	-	682 (97.0%)	284 (94.7%)	0.105
	+	21 (3.0%)	16 (5.3%)	
Atrial fibrillation	-	691 (98.3%)	299 (99.7%)	0.124
	+	12 (1.7%)	1 (0.3%)	
COPD	-	695 (98.9%)	298 (99.3%)	0.732
	+	8 (1.1%)	2 (0.7%)	
Cerebrovascular disease	-	678 (96.4%)	288 (96%)	0.874
	+	25 (3.6%)	12 (4%)	
Smoking	-	579 (82.4%)	253 (84.3%)	0.504
	+	124 (17.6%)	47 (15.7%)	
Drinking	-	634 (90.3%)	22 (7.3%)	0.283
	+	68 (9.7%)	195 (65.2%)	
Hgb, g dL^−1^		131.0 (114.0, 145.0)	133.00 (115.0, 146.5)	0.207
ALB, g L^−1^		37.4 ± 4.7	37.7 ± 4.0	0.340
BUN, mg dL^−1^		5.12 (4.2, 6.1)	5.16 (4.1, 6.3)	0.479
SCR, mg dL^−1^		63.2 (54.1, 73.4)	63.3 (55.0, 73.8)	0.268
LVEF		66.92 (64.4, 70.0)	67.39 (64.7, 70.0)	0.975
LVFS		37.21 (35.3, 39.6)	37.21 (35.4,37.2)	0.962
PO_2_		70.90 (65.4, 77.1)	70.90 (66.2, 78.0)	0.185
FVC % pred		111.4 ± 17.9	110.4 ± 17.6	0.473
FEV1% pred		103.0 (92.0, 116.4)	103.0 (89.0, 115.0)	0.621
FEV1/FVC % best		76.0 (70.3, 80.3)	75.2 (70.9, 81.0)	0.692
PEF % pred		106.0 (90.0, 117.0)	104.00 (89.0, 117.0)	0.419
Tumor site	RH	104 (14.8%)	44 (14.7%)	0.812
	LH	50 (7.1%)	27 (9%)	
	LAR	424 (60.3%)	175 (58.3%)	
	SR	40 (5.7%)	12 (4%)	
	IR	3 (0.4%)	2 (0.7%)	
	TC	1 (0.1%)	1 (0.3%)	
	APR	56 (8%)	29 (9.7%)	
	Other	25 (3.6%)	10 (3.3%)	
Surgical route	Laparoscope	625 (88.9%)	266 (88.7%)	0.999
	Open	78 (11.1%)	34 (11.3%)	
TNM	I-II	404 (57.5%)	170 (56.7%)	0.454
	III	243 (34.6%)	112 (37.3%)	
	IV	56 (8%)	18 (6%)	
Operation time, min		218.0 (182.5, 262.0)	225.0 (191.0, 268.0)	0.102
Anesthesia time, min		258.0 (216.0, 298.0)	258.0 (224.5, 303.5)	0.295
Crystalloids, mL		2000.0 (1500.0, 2500.0)	2000.0 (1775.0, 2500.0)	0.684
Synthetic colloids, mL		0.0 (0.0, 500.0)	0.0 (0.0, 500.0)	0.981
Urine output, mL		400.0 (200.0, 600.0)	500.0 (300.0, 700.0)	0.066
Blood loss, ml		100.0 (50.0, 100.0)	100.0 (90.0, 125.0)	0.101
RBCT	-	645 (91.7%)	273 (91%)	0.790
	+	58 (8.3%)	27 (9%)	

Data were presented as mean ± standard deviation or median (the 25% percentile, the 75% percentile) for continuous variables and count (percentage) for categorical variables. -, No; +, Yes

*ICU* Intensive care unit, *BMI* Body mass index, *CCI* Charlson comorbidity index, *ASA* Classification, American Society of Anesthesiologists physical status classification, *mFI* Modified frailty index, *NRS2002* Nutritional risk screening 2002, *PCI* Percutaneous transluminal coronary intervention, *COPD* Chronic obstructive pulmonary disease, *Hgb* Hemoglobin, *ALB* Serum albumin, *BUN* Blood urea nitrogen, *SCR* Serum creatinine, Creatinine, *LVEF* Left ventricular ejection fraction, *LVFS* Left ventricular fraction shortening, *PaO*_*2*_ Arterial oxygen saturation, *FVC* Forced vital capacity, *FEV1* Forced expiratory volume in 1 s, *FEV1/FVC* Forced expiratory volume in 1 s / Forced vital capacity, *PEF* Peak expiratory flow, *RH* Right hemicolectomy, *LH* Left hemicolectomy, *LAC* Low anterior resection, *SR* Sigmoid resection, *IR* Ileocecal resection, *TC* Transverse colectomy, *APR* Abdominoperineal resection, *TNM* Classification of malignant tumours, *RBCT* Red blood cell transfusion

### Imputation of missing data

Following multiple imputation, all predictors in the data showed total effective rates of FVC 95.5% (958/1003), FEV1% 95.5% (958/1003), FEV 1/FVC 95.5% (958/1003), PEF 95.5% (958/1003). There were no significant differences between the reference data and the imputed dataset. The imputed datasets were employed for all cohort analyses. Postoperative patient locations were complete in both datasets. Consequently, the regression analysis data were aligned with the identified eligible patient count. Supplementary Table S1 provides details of missing data.

### Screening predictors

**Fig. 2:**
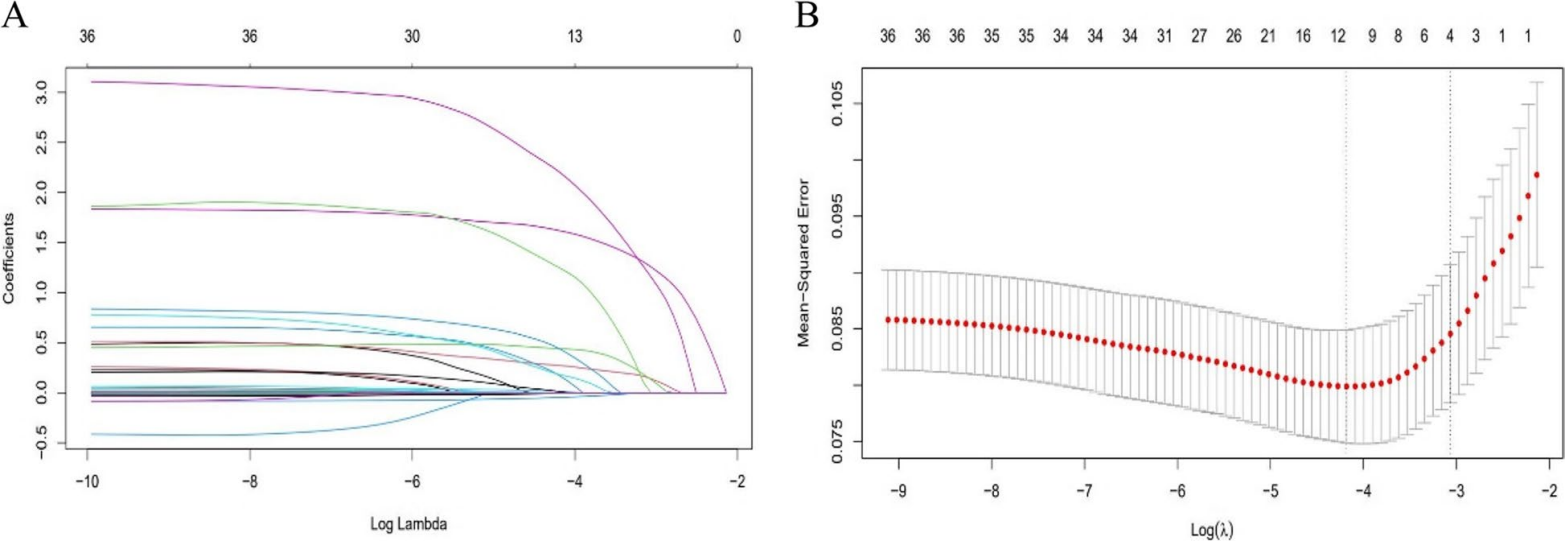
Predictors selection using the LASSO logistic regression model. **A** LASSO coefficient profiles of the 36 predictors. A coefficient profile plot was plotted against the log(λ) sequence, and the 12 non-zero coefficients were chosen at the values selected using tenfold cross-validation. **B** Optimal parameter (λ) selection in the LASSO model used tenfold cross-validation and minimum criteria. The partial likelihood deviance (binomial deviance) curve was plotted vs. log(λ). Dotted vertical lines were drawn at the optimal values by using the minimum criteria and the 1 SE of the minimum criteria (the 1-SE criteria)

Screening of the 36 variables in the training cohort using the Least Absolute Shrinkage and Selection Operator (LASSO) method identified 12 predictors with non-zero coefficients at the minor standard error (Fig. 2). These predictors included age, CCI, ASA, NRS2002, coronary heart disease, percutaneous coronary intervention, atrial fibrillation, COPD, serum albumin, LVEF, and surgical approach. All predictors were subsequently subjected to multivariate analysis. Multiple logistic regression analysis disclosed seven variables as independent predictors within the model: age (*P* = 0.003, odds ratio [OR] 3.623, 95% confidence interval [CI] 1.535–8.551), NRS2002 (*P* = 0.000, OR 6.129, 95% CI 2.920–12.863), ALB (*P* = 0.013, OR 0.921, 95% CI 0.863–0.982), atrial fibrillation (*P* = 0.000, OR 20.017, 95% CI 4.191–95.609), COPD (*P* = 0.009, OR 8.151, 95% CI 1.674–39.676), FEV1/FVC (*P* = 0.040, OR 0.966, 95% CI 0.935–0.998), and surgical procedure (*P* = 0.024, OR 0.425, 95% CI 0.202–0.891), as depicted in Table 2.

**Table 2:** Risk factors for postoperative ICU admission following radical resection for colorectal cancer

Risk Factors	OR	95% CI	*p*-Value
Age, years			
≤ 64	Ref		
65–74	3.62	1.535–8.551	0.000
≥ 75	2.83	1.082–7.405	0.034
CCI (vs. < 3)	2.36	0.907–6.122	0.078
ASA			
II	Ref		
III	1.17	0.597–2.271	0.655
IV	1.56	0.267–9.400	0.612
NRS2002 (vs. < 3)	6.13	2.920–12.863	0.000
Coronary heart disease (vs. No)	1.56	0.734–3.286	0.249
PCI (vs. No)	1.56	0.472–5.337	0.456
Atrial fibrillation (vs. No)	20.02	4.191–95.609	0.000
COPD (vs. No)	8.15	1.674–39.676	0.009
Serum albumin, g L^−1^	0.92	0.863–0.982	0.013
LVEF	0.98	0.943–1.008	0.139
FEV1/FVC	0.97	0.935–0.998	0.040
Surgical route (vs. Laparoscope)	2.36	1.202–0.891	0.024

*OR* Odds ratio, *CI* Confidence interval, *CCI* Charlson comorbidity index, *ASA* Classification, American Society of Anesthesiologists physical status classification, *NRS2002* Nutritional risk screening 2002, *PCI* Percutaneous transluminal coronary intervention, *COPD* Chronic obstructive pulmonary disease, *LVEF* Left ventricular ejection fraction, *FEV1* Forced expiratory volume in 1 s, *FEV1/FVC* Forced expiratory volume in 1 s/ Forced vital capacity

### Development and validation of nomograms for risk prediction

**Fig. 3:**
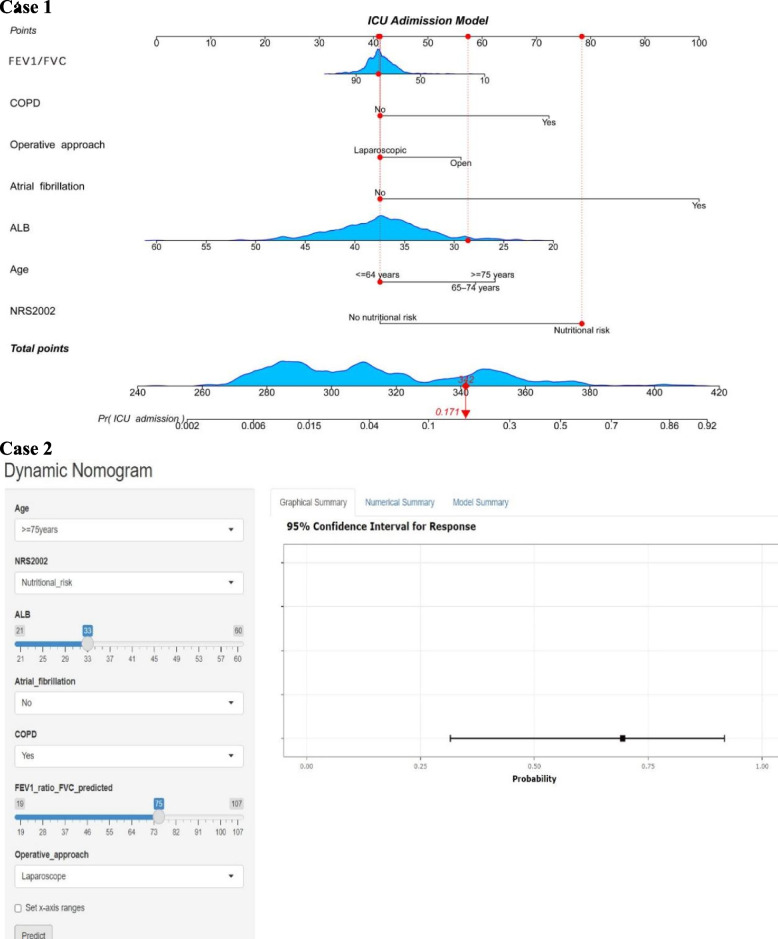
Nomogram for predicting postoperative ICU admission. Oxycodone refers to receiving oxycodone before the end of surgery. Case 1 represents a patient who underwent radical resection of colorectal cancer surgery; the patient had a FEV1 / FVC of 83%, no chronic obstructive pulmonary disease, underwent laparoscopic surgery, no atrial fibrillation, preoperative serum albumin 28 g/L, age of 64 years or less, and had nutritional risk. In consideration of these seven variables, the total score for this patient was 342, and the probability of postoperative ICU admission was 0.171 (17.1%). Case 2 represents an online dynamic nomogram at https://picuadmission.shinyapps.io/DnamicNomogram/, depicting an example for predicting the probability of postoperative ICU admission for a 75 years old patient or more with nutritional risk, preoperative serum albumin 32 g/L, no atrial fibrillation, chronic obstructive pulmonary disease, FEV1 / FVC of 75% and underwent laparoscopy surgery. ICU, intensive care unit; FEV1/FVC, Forced expiratory volume in 1 s / Forced vital capacity; COPD, chronic obstructive pulmonary disease; ALB, Serum albumin; NRS2002, Nutritional Risk Screening 2002

The seven predictors from the aforementioned logistic regression model were incorporated into the nomogram (*R*^2^ = 0.382, C-index = 0.873) (Fig. 3). For each patient, a higher total score indicated a elevated risk of postoperative special ICU admission following radical or palliative CRC surgery. For instance, consider a patient with an FEV one-second rate of 83%, absence of chronic obstructive pulmonary disease, laparoscopic surgery, atrial fibrillation, preoperative serum albumin 28 g/L, age 64, and nutritional risk. The corresponding scores would be approximately 41, 41, 41, 41, 58, 41, and 79 points, respectively. The cumulative score totaled around 342 points, suggesting an estimated 17% likelihood of postoperative mandatory ICU admission. The Hosmer–Lemeshow test (H–L test) showed a good model fit (*P* = 0.367).

### Predictive accuracy and net benefits of nomograms

**Fig. 4:**
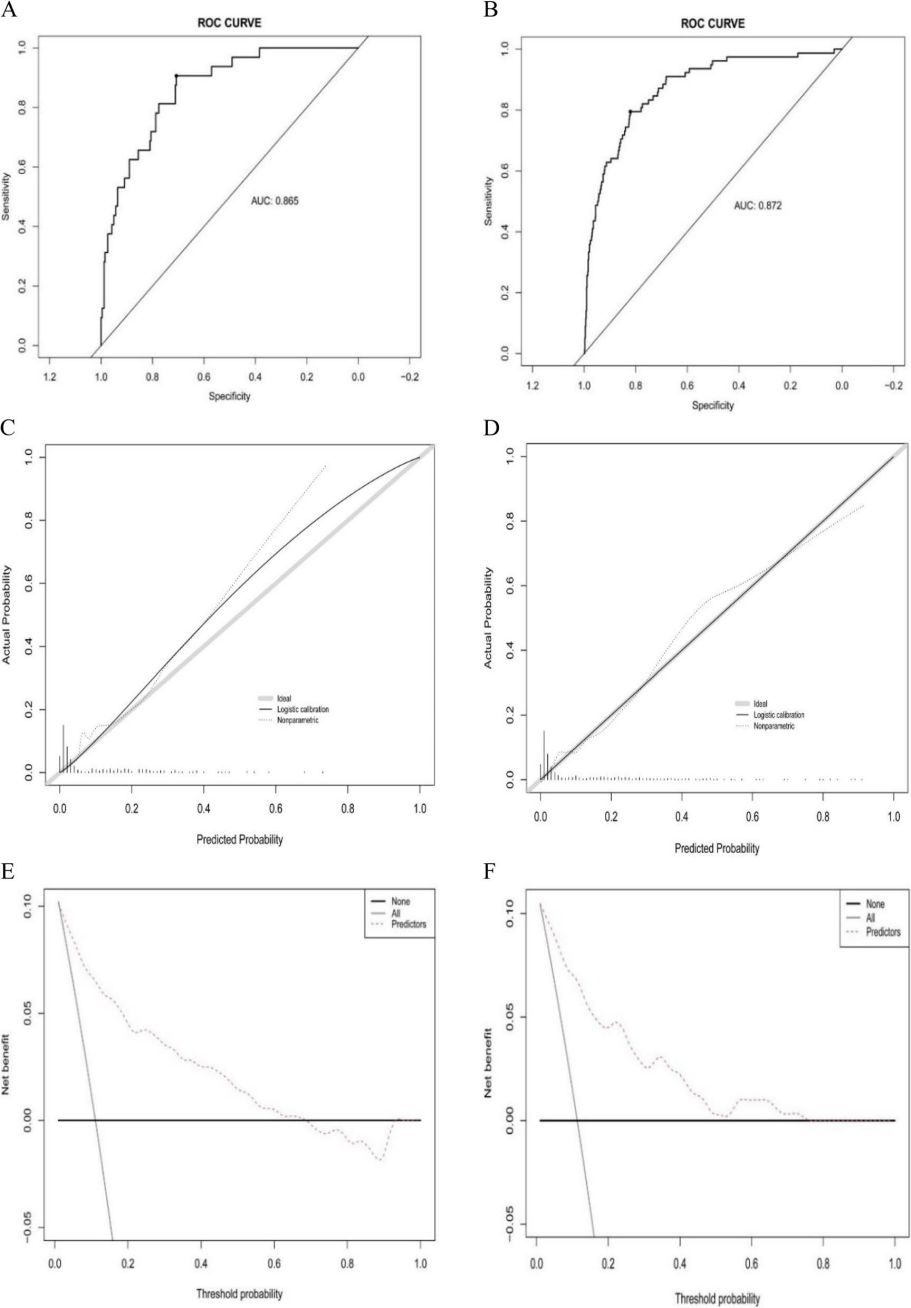
ROC curves. **A** Training cohort. **B** Validation cohort. Calibration curve for predicting probability of postoperative ICU admission. **C** Training cohort. **D** Validation cohort. Decision curve analysis in prediction of postoperative ICU admission. **E** Training cohort. **F** Validation cohort. ROC, receiver operating characteristic; AUC, area under the ROC curve; ICU, intensive care unit

In the training cohort, the AUC was 0.865 (Fig. 4A), with the calibration curve closely aligning with the ideal diagonal (Fig. 4C). Additionally, DCA illustrated notably superior net gains in the predictive model (Fig. 4E). A validation cohort comprising 300 patients evaluated the nomogram. The AUC was 0.872 (Fig. 4B), surpassing that of the training cohort, underscoring the nomogram's outstanding accuracy. Similarly, the calibration curve of the validation cohort closely resembled the ideal diagonal (Fig. 4D), signifying strong agreement with the model. Moreover, DCA exhibited significant net benefit for both the predictive model and the validation cohort (Fig. 4F). Collectively, these findings highlight the predictive nomogram's exceptional potential for informing clinical decision-making.

### Online tool for nomograms

The nomogram, derived from LASSO and multiple regression analysis, serves as a simplified tool for clinicians, capable of predicting postoperative ICU admission based on various combination of key predictor variables. To enhance accessibility for researchers and clinicians, we developed a user-friendly online web server https://picuadmission.shinyapps.io/DnamicNomogram/. Users can input the seven predictive factors into the model, generating predicted probabilities, thereby enhancing clinical utility (Fig. 3).

### Risk stratification with the risk prediction Nomogram

Utilizing the receiver operating characteristic (ROC) curve of the risk prediction nomogram, optimal diagnostic sensitivity (79.5%) and specificity (82.1%) were attained, corresponding to a risk prediction value of 16.4%. Consequently, a risk prediction value > 16.4% delineates the high-risk group for postoperative ICU admission following colorectal cancer surgery; conversely, a prediction value ≤ 16.4% identifies the low-risk group (Table 3).

**Table 3:** Diagnostic performance of the training and validation cohorts

Variable	Training cohort	Validation cohort
Area under ROC Curve	0.872	0.865
Sensitivity (%)	79.5	90.6
Specificity (%)	82.1	70.7

*ROC* Receiver operating characteristic

## Discussion

This is the first study to use preoperative and intraoperative factors to guide clinical decisions associated with ICU admission after radical or palliative surgery for CRC. The results of the study indicated that patient age, preoperative serum albumin level, nutritional risk screening, atrial fibrillation, chronic obstructive pulmonary disease, surgical approach, and the FEV one-second rate were independent predictors of ICU admission after radical or palliative surgery for CRC. In situations where ICU resources are limited, the identification of high-risk patients in postoperative intensive care can assist clinicians with decisions and arrangements for postoperative intensive care, thus reducing unnecessary waste of resources and complications caused by ICU admission.

Age is a significant predictor of postoperative complications in cardiac and noncardiac surgery. Patients with CRC who are elderly and may have comorbidities are more prone to developing postoperative complications, having a higher risk of postoperative death, and being more likely to require ICU treatment after surgery. A previous study showed an association between advanced age and early ICU admission after lower gastrointestinal surgery [21]. Another study found that routine overnight stays in the ICU for CRC patients aged 80 years and older after surgery can reduce the risk of adverse postoperative outcomes [22]. Due to the increasing number of elderly patients with CRC, the disease burden associated with advanced age has become a public health problem. Therefore, we foresee that more patients with CRC who are elderly and have comorbidities may require specific treatment in the ICU after surgery.

The FEV one-second rate emerges as an independent predictor of immediate postoperative ICU admission, thereby integrated into the predictive model. This is the first study to focus on preoperative pulmonary function tests for guiding clinical decisions regarding postoperative ICU admission. Preoperative assessment of cardiopulmonary function is essential, as anesthesia and surgical factors can affect the respiratory and circulatory systems. Patients with poor preoperative lung function are more likely to require ICU respiratory support after general anesthesia. Spirometry has been widely used to assess lung function before thoracic surgery [23], and its usefulness in non-thoracic surgery has also been demonstrated [24, 25]. However, a recent meta-analysis found that due to differences in the design of various studies, the ability of pulmonary function tests to predict postoperative pulmonary complications before non-thoracic surgery remains to be confirmed [26].

The present study also found that preoperative nutritional risk in patients was an independent predictor of immediate postoperative ICU transfer. Patients who were undernourished before surgery often showed delayed postoperative recovery and greater respiratory muscle weakness than others. Lee et al. observed a close relationship between preoperative malnutrition and the occurrence of postoperative respiratory failure in patients undergoing colorectal resection [27]. This may indicate a higher risk of requiring postoperative mechanical ventilation in malnourished patients, but whether the postoperative provision of appropriate ventilatory support to these patients will control potential respiratory disorders, reduce postoperative complications, and support the recovery of underlying diseases still requires further investigation.

Serum albumin levels were also shown to be associated with postoperative mechanical ventilation for more than 48 h, reintubation, myocardial infarction, cardiac arrest, lung infection, wound infection, septic shock, and deep-vein thrombosis in patients with CRC [28], as well as an increased risk of pulmonary embolism, return to the operating room, prolonged hospital stay, and increased 30-day mortality [29–31]. The Nutritional Risk Screening 2002 (NRS 2002) emerges as an effective, reliable, and well-validated tool for assessing malnutrition, despite its previous lack of use as a predictor of immediate postoperative ICU transfer [32].

The presence of malnutrition is associated with major cardiovascular events, acute renal failure, infections, increased hospital costs, and mortality [33]. Compared to other tumor types, CRC patients exhibit the highest malnutrition rates [34, 35], necessitating intensified postoperative monitoring and treatment in the ICU for severely malnourished individuals.

The susceptibility of CRC patients to malnutrition stems from various factors, including the tumor itself and treatment-related side effects. While some patients can maintain adequate nutrition, others experience malnutrition. Therefore, addressing the use of neoadjuvant chemotherapy (NACT) in this cohort is crucial. While NACT may hold promise in improving the nutritional status of specific patients, its precise impact remains debatable, especially regarding its ability to predict postoperative ICU admission [36]. Further investigation into the role of NACT in managing the nutritional needs of patients with CRC is warranted.

The presence of AF and COPD before surgery was found to be independent predictors of immediate ICU transfer in patients undergoing radical resection of CRC. A large national cohort study in the United States found that preoperative AF and increased risk of early cardiovascular complications post-noncardiac surgery [37]. AF precipitates several cardiovascular sequelae, including reduced stroke volume, increased pulmonary artery pressure, and heightened susceptibility to tachycardia, along with predisposition to myocardial ischemia, fluid overload, and respiratory failure [38–41]. Additionally, COPD independently correlates with postoperative complications and mortality, encompassing potential complications like pneumonia, respiratory failure, myocardial infarction, and sepsis [42, 43]. However, not all patients with AF or COPD should be admitted to the ICU after surgery instead of being extubated immediately. We emphasize that in certain cases, especially in patients with significant cardiopulmonary diseases, closer monitoring and care may be necessary, and may potentially require transfer to the ICU to determine the optimal extubation timing.

Open surgery, encompassing both planned procedures and cases where laparoscopic surgery was converted to open surgery due to intraoperative complexities, was found to be a significant risk factor for immediate postoperative ICU admission in patients with CRC. In recent decades, laparoscopic surgery has gradually replaced open colorectal surgery due to its associations with reduced trauma, postoperative pain, infectious complications,blood loss, and recovery time. The rates of local recurrence, disease-free survival, and overall survival are similar to those of open surgery [43–45]. Therefore, patients who undergo laparoscopic surgery recover more quickly than those who undergo open surgery, and the need for postoperative ICU support is less. Despite this, surgeons may resort to open procedures for cases involving anticipated complexity or unforeseen challenges that occur intraoperatively. However, the present study did not investigate CRC-specific risk factors, which are similar to those identified by Pan et al. in surgery for gastric cancer, in which an investigation into the risk factors for postoperative ICU admission in patients after gastric cancer surgery identified combined organ resection as a risk factor [7]. However, they did not find specific risk factors that were unique to gastric cancer surgery. This suggests that the characteristics of specific surgical procedures for gastrointestinal tumors may not be the determining factor for predicting postoperative ICU admission risk in these patients.

In summary, this retrospective study evaluated predictors in the pre-and intraoperative patient trajectory to predict the risk of ICU admission in patients receiving either curative or palliative surgery for CRC. The predictive online website provides a simple, intuitive, convenient, and practical predictive tool for clinical use. Not only can clinicians view the nomogram, but they can also utilize the website to calculate the risk prediction value for patients' postoperative mandatory ICU admission, thereby identifying high-risk individuals who may benefit from early intervention. The strength of this study was the sufficient sample size. Additionally, a series of preoperative and intraoperative predictive factors were incorporated into the model. The model focused not only on preoperative indicators of cardiopulmonary function but also on risk factors associated with nutrition and frailty. Thus, the indicators and risk factors provided a comprehensive reflection of the physical status of the patient.

### Limitations

The study had several limitations. Firstly, it was a single-center investigation, and the results were only internally validated. In the future, it is necessary to conduct multicenter investigations with external validation to enhance the reliability and generalizability of the current findings. Secondly, given the retrospective nature of this study, some degree of internal bias is inevitably present. Thirdly, the study focused on patients who were admitted to the ICU immediately after surgery, excluding those who developed complications requiring ICU admission beyond three days after surgery. While this exclusion may introduce bias, the proportion of such patients in the study was minimal. Moreover, the potential benefits of immediate postoperative ICU admission for patients with CRC may outweigh the considerations for those developing delayed postoperative complications necessitating ICU readmission. Fourthly, multiple imputation was used to address the issue of missing data. Although this may have introduced some degree of bias, considering that the amount of missing data was less than 5% of the total sample size and was random, the bias introduced by imputation would be expected to be relatively small.

## Conclusion

The present study generated a clinical predictive model and an online predictive website with good capabilities based on the identified predictors of specific ICU admission following radical CRC surgery. The identified predictors included age, preoperative serum albumin level, nutritional risk screening, atrial fibrillation, COPD, FEV1/FVC, and the surgical approach. The use of the nomogram and online tool to evaluate the need for postoperative ICU admission will not only provide the maximum benefit to patients but will also provide a reference for the rational distribution of medical resources, improve the quality of medical care, and promote patients’ recovery.

### Supplementary Information

Supplementary Material 1.

## Data Availability

The datasets used and analyzed during the current study are available from the first author on reasonable request.
