# Supplementary material for: Construction and validation of a risk prediction model for postoperative ICU admission in patients with colorectal cancer: clinical prediction model study
Source: BMC Anesthesiol. 2024 Jul 4;24:222. doi: 10.1186/s12871-024-02598-3 (PMC11223334; doi:10.1186/s12871-024-02598-3)
Supplement: Supplementary file 1 — Supplementary Material 1. [file 12871_2024_2598_MOESM1_ESM.docx]

**Supplementary Table S1.** Missing data.

| Characteristic | Missing Values,  n (%) | Value  Median (SD) | P value |
| --- | --- | --- | --- |
| FVC | 45(0.05) | 110.99 (18.04) | 0.954 |
| FEV1 | 45(0.05) | 102.97 (20.07) | 0.990 |
| FEV1/FEV | 45 (0.05) | 74.68 (9.91) | 0.749 |
| PEF | 45 (0.05) | 104.22 (23.20) | 0.877 |

SD, Standard deviation; FVC, Forced vital capacity. FEV1, Forced expiratory volume in 1 second; FEV1/FVC, Forced expiratory volume in 1 second/ Forced vital capacity; PEF, Peak expiratory flow.
